# The Construct Validity of the German Academic Self-regulation Questionnaire (SRQ-A) within Primary and Secondary School Children

**DOI:** 10.3389/fpsyg.2017.01032

**Published:** 2017-06-22

**Authors:** Julia Kröner, Christina Goussios, Caroline Schaitz, Judith Streb, Zrinka Sosic-Vasic

**Affiliations:** ^1^Department of Psychiatry and Psychotherapy III, University Clinic of UlmUlm, Germany; ^2^Department of Forensic Psychiatry and Psychotherapy, University of UlmUlm, Germany

**Keywords:** questionnaire, validation, self-determination theory, self-reguated learning, motivation, children (9–14), school, measurement

## Abstract

The assessment of students' motivation can be a powerful tool in enhancing and understanding students' learning. One valid and often applied self-report measure is the Academic Self-Regulation Questionnaire (SRQ-A) which is grounded in the self-determination theory. However, to date, there is still no German equivalent to the English version of this questionnaire. Therefore, the aim of the present study was to adapt and validate the SRQ-A on a representative German student sample, consisting of 672 children (327 girls), ages 8–14 from one primary and two secondary German schools. First, the translation-back-translation method was used to ensure the linguistic equivalence of the German questionnaire. Second, item analysis of the generated scores of the German SRQ-A were conducted. Third, the multidimensional factorial structure of the original measure was tested with confirmatory factor analysis (CFA) using maximum likelihood estimation. Last, additional construct validity of the German SRQ-A was tested using correlational analyses with convergent and divergent measures. After conducting CFA, four items were excluded from the original questionnaire, due to loadings lower than 0.40, resulting in 28 items. The German SRQ-A showed good internal consistency for all subscales, with Chronbach's α ranging between 0.75 and 0.88. The simplex-structure of the original measurement could also be confirmed, however, the four-factorial model could not be replicated. The measurement showed good convergent and discriminant validity with other related questionnaires. In summary, the German SRQ-A is a reliable and valid self-report instrument for the assessment of self-determined motivational styles within the school context.

## Introduction

Within the academic context, motivational abilities are frequently considered by parents and teachers to be one of the most crucial factors explaining the child's scholastic success. Several influential approaches to motivation have been established, of which the Self-Determination Theory (SDT) according to Deci and Ryan (1985, 2000) is one the most influential concepts with an impressive empirical background. Two fundamental claims ground this theory: First, different types of motivation—or more broadly spoken—behavioral regulation are postulated with respect to the degree they represent autonomous or self-determined (vs. controlled) functioning on a continuum from low—i.e., external regulation and introjected regulation—to high values of self-regulation—i.e., identified regulation and intrinsic regulation. According the theoretical framework, intrinsic motivation is the most self-determined motivational style. It is defined as the motivational style in which children engage in an activity because of interest and enjoyment, rather than because of external incentives. Next, there is identified regulation. Children, who display this type of motivational style have found the behavior to be personally important, and have therefore accepted it as their own. The two other forms of regulation styles, namely, introjected and external regulation are less self-determined as the previously described motivational styles. Children, who experience introjected regulation complete an activity, because they feel internal pressures—such as shame or guilt—to fulfill the task. The least self-determined motivational style is external regulation. Those children, who have adapted this kind of motivational style perform because they feel externally pressured or they receive external incentives, i.e., rewards or punishments. All four motivational styles are, even though considered as individual differences, not treated as “trait” concepts due to a lack of generality or stability. At the same time, there are not treated as “state” concepts, as they are considered to be more stable than typical states which show stronger fluctuation over time and situation. Second, high levels of autonomous self-determined motivation are considered “to be fostered by the experience of three fundamental basic psychological needs—i.e., the need for autonomy (experiencing a sense of volitional and psychological freedom), the need for competence (experiencing personal effectiveness), and the need for interpersonal relatedness (experiencing closeness and mutuality in interpersonal relationships)” (Sosic-Vasic et al., 2015, p. 2). In contrast, if those needs are experienced less frequently, it might result in a decrease of motivation and overall well-being, ultimately leading to less self-determined behavior. Following SDT, there are various positive outcomes of self-determined motivation styles on learning. For example, teachers who are providing a high amount of autonomy support have students who demonstrate higher engagement during classes, higher efforts, and higher interest for the subject taught (Reeve et al., 2002; Tsai et al., 2008). Furthermore, past research indicates that over time, students who have an available scope of action to choose from during the learning process, choose tasks that are optimally demanding, instead of choosing tasks that are too simple for the student's educational capacities (Boggiano et al., 1988). Furthermore, various studies demonstrated that autonomous learning is associated with higher scholastic achievement (e.g., Grolnick et al., 1991; Fortier et al., 1995; Guay and Vallerand, 1997). For example, the more independent the students are learning, the better their grades and the higher the experienced self-efficacy. Autonomous learning is also associated with deeper mental processing and better memory capacities (Benware and Deci, 1984; Vansteenkiste et al., 2004), whereas extrinsically motivated learning is hindering creative cognitive processes (Amabile, 1995). In addition, students' emotional experiences are positively influenced by the gratification of the basic needs. Studies showed that teachers, who are utilizing autonomy supporting behaviors, have students who experience more positive feelings in class and during the learning process (e.g., Vallerand et al., 1989). However, students with teachers who are highly controlling, demonstrate higher anxiety (Assor et al., 2005). A more recent study indicated that intrinsic motivation correlated with psychological well-being in primary school children (Burton et al., 2006). Furthermore, there are positive effects of considering the students' basic needs within the context of interpersonal relatedness: Students, who are highly interpersonally related tend to accept scholastic behavioral rules better than their less related peers (Tsai et al., 2008). Additionally, students who feel strongly socially integrated, display a stronger feeling of school affiliation (Allen, 2003). Finally, some studies have investigated gender differences regarding basic needs and self-determined motivational styles and have revealed rather inconsistent results. For example, while Grolnick and Ryan (1990) showed that girls felt more autonomous than boys, other investigations did not reveal significant differences (Deci et al., 1992).

Accordingly, identifying students' self-determined motivation styles might be a necessary step for both, researchers and professionals in educational practice. However, only a few well-designed and established questionnaires are available measuring motivational styles within an academic context. Among those, of high relevance is the Motivational Styles of Learning Questionnaire (MSLQ; Pintrich and DeGroot, 1990) which is based on the Self-Regulation Learning Model (SRL-Model) according to Pintrich and DeGroot (1990). In general, the SRL-Model consists of three different cognitive strategies, which are influencing, controlling, and regulating the students' learning process (e.g., Pintrich, 1989, 1999; Garcia and Pintrich, 1994). The first set of strategies consists of cognitive learning components, such as organization, elaboration, and rehearsal, which are related to the student's performance in an academic context (e.g., Pintrich, 1989; Pintrich and DeGroot, 1990). The second set of strategies is concerned with the students' metacognition and self-regulation of cognition—i.e., strategies that are used to monitor, plan, and regulate cognitive activities (e.g., Pintrich, 1988a,b; Pintrich et al., 1999). Lastly, the third set of cognitive strategies includes strategies to manage various resources, such as controlling environmental factors (e.g., teachers, peers, time, and effort; Ryan and Pintrich, 1998; Pintrich, 1999). Finally, various studies have stressed the importance of incorporating motivational components as well as goal orientation into the SRL-Model, as they have shown to influence academic performance and relate to cognitive components of learning (e.g., Wolters et al., 1996; Pintrich, 1999). Accordingly, the MSLQ (Pintrich and DeGroot, 1990), assesses not only motivational but also cognitive processes. However, the instrument was designed to be implemented with post-secondary students and results from its first large empirical investigation as included in the manual are based on college students (Pintrich et al., 1991). The other highly relevant measure is the Academic Self-Regulation Questionnaire (SRQ-A; Ryan and Connell, 1989) which focuses on motivational styles based on the SDT. In contrast to the MSLQ, the SRQ-A has been developed for children in primary and secondary school, beginning at third grade, and thus represents one of the most prominent and widely used questionnaires within the school context.

This self-report measurement assesses individual differences in motivational styles or behavioral regulation by asking the reasons as to why children complete their school work. The questionnaire consists of 26 items and asks for the reasons as to why the respondent displays a certain behavior (*Why do I do my homework? Why do I work on my classwork? Why do I try to answer hard questions in class? Why do I try to do well in school?*). It also provides various possible preselected reasons that represent different motivational styles—i.e., intrinsic motivation, identified regulation, introjected regulation, and external regulation. Due to this, some of the items mention the same reason but refer to different behaviors. Respondents rate each of the provided reasons to perform on a four-point Likert-scale with response options ranging from 1 (*not at all true*) to 4(*very true*); thus, higher scores indicate a higher degree of agreement. Psychometric analyses of the original SRQ-A questionnaire revealed a two-factorial structure of the items, ranging on opposing ends of an autonomy spectrum. However, despite the fact that this structure would have meaningful discriminant validity, the authors decided to include a four-factorial structure, in order to account for psychological meaningfulness of the categories, which would have been lost due to the Procrustes bed of the utilized factorial analytical approach (Ryan and Connell, 1989). The upon implemented analysis then demonstrated moderate to high levels of internal consistency for the four subscales ranging from 0.62 to 0.82 (Ryan and Connell, 1989). The final questionnaire consists of 26 items (out of the implemented 34 items). Although, the SRQ-A has been translated into various languages (e.g., Alivernini et al., 2011; Bagceci and Kanadli, 2014; Pichardo et al., 2014) and has been validated psychometrically and qualitatively, to the best of our knowledge, there is no equivalent German validation study of the SRQ-A. One research group has translated and adapted the SRQ-A to German (Müller et al., 2007; Gnambs and Hanfstingl, 2015). However, despite the importance of these results, this German translation does not allow for measuring primary school children, because the authors have adapted their German translation of the SRQ-A to fit an adolescent German target population starting at the ages 10 and up. Furthermore, those authors have only implemented 9 out of 26 items from the original SRQ-A in their German version, while also adding several items from other scales such as the Academic Motivation Scale (Vallerand et al., 1992), resulting in a 16-item questionnaire that is not an equivalent translation of its original English counterpart. Another German translation of the SRQ-A stems from Wild and Krapp (1995). However, even though this German version was already used in several studies, the psychometric properties of its translation have never been investigated and the questionnaire has never been validated (Levesque et al., 2004). Therefore, it is warranted to evaluate the psychometric qualities of the SRQ-A also with primary school children, as this questionnaire was designed to be used in a broad academic context.

Considering the need of validated measures to advance our knowledge of self-determined motivation styles in different cultures we consider the translation and belonging psychometric evaluation and validation as mandatory for responsible usage in psychological research. Thus, the present study aims to provide a German version of the SRQ-A, which is suitable to measure self-determined motivational styles in primary school children attending 3rd grade or higher, and to assess its psychometric properties in order to ensure the equivalence to the English original. Consequently, the purpose of the present study was: (a) To translate the SRQ-A into German with the most comparable fit to the original target group aged 8 years and older, (b) To complete an item analysis of scores generated by the German SRQ-A, (c) to test the multidimensional factorial structure proposed by the original measure with a confirmatory factor analysis (CFA), (d) to test the convergent and divergent validity of the translated version by assessing relations to other measures in a large sample of German primary and junior high school children. Since previous findings reported rather inconsistent findings regarding gender differences with respect to motivational styles, the previous study aims to report additionally gender specific findings.

## Materials and methods

### Participants

In a cross-sectional study we investigated 672 children. The mean age in the sample was 10.27 years (*SD* = 1.28), and 48.6% of the sample were girls (*N* = 327). 284 children attended primary school (Grundschule; 42.2%), 223 Middle school (33.1), and 165 Gymnasium (24.5%; for a detailed sample description see Table 1). The German school system has a unique structure: After finishing primary school, children are separated into two different levels of schooling—Middle school and Gymnasium—based on their grades. 133 children were third graders (19.8%), 153 children fourth graders (22.7%), 220 children fifth graders (32.79%), and 167 children sixth graders (24.8%). 15 participants were excluded from data analysis, due to missing responses on at least one item of the SRQ-A. For detailed sample description see Table 1.

**Table 1:** Amount of children by level of schooling.

**Level of Schooling**	***N***	**%**	**Age (Mean)**	**Age (*SD*)**	**Age (Range)**	**Boys**	**%**	**Girls**	**%**
Total	672	100.0	10.27	1.29	8–14	344	100.0	327	100.0
Primary school	284	42.2	9.11	0.81	8–11	151	43.9	132	40.4
Middle school	223	33.1	11.24	0.84	10–14	110	32	113	34.6
Gymnasium	165	24.5	10.97	0.78	9–13	83	24.1	82	25.1

*N, Number of Students; %, Percent of sample (total, boys, girls); SD, Standard Deviation*.

### Procedure

#### Participant recruitment

Schools were contacted by project staff and asked to participate in the study. After the respective school's dean consented to partake in the study, we initiated an information session for children, parents, and teachers, upon which the child and their respective parent volunteered to participate. Both, parental and child written consent was obtained prior to data collection. Children attended regular school hours, during which they were questioned, in order to avoid additional use of time. The study was approved by the local Internal Review Board of the Medical Faculty of the University of Ulm, according the Declaration of Helsinki.

#### Back-translation

The study comprised of two stages: Translation of the instrument into German and its subsequent validation. The systematic back-translation technique was used to ensure that the original meaning of the SRQ-A was not altered. The original English version of the scale was translated into German independently by two bilingual speakers. A team of psychologists with expertise in the subject of SDT reviewed the translations. Based on the translations and the questions raised by the research team, we optimized the German version of the questionnaire.

The German version of the SRQ-A was then back-translated independently by two different bilingual speakers to ensure the conceptual equivalency to the original version. Subsequently, the research team and all translators compared the back-translation with the original version to identify any questions that were not equivalent or problematic.

#### Data collection

After all of the researchers and translators reached an agreement of the appropriateness of the translated items, a series of pre-tests evaluating our German translation were conducted with a total of 59 children from primary and secondary school. This procedure allows for the assessment of the amount of understanding achieved by the translated questionnaire (Sosic-Vasic and Streb, 2010; Sosic-Vasic et al., 2015). The results obtained by the conducted pre-tests demonstrated good psychometric properties. Cronbach's α ranged from 0.77 to 0.87 (intrinsic motivation: α = 0.87; identified regulation: α = 0.83; introjected regulation: α = 0.83; external regulation: α = 0.77). The item-difficulty of the German version of the SRQ-A ranged between 0.36 and 0.83 (intrinsic motivation: 0.36 to 0.71; identified regulation: 0.74 to 0.82; introjected regulation: 0.57 to 0.83; external regulation: 0.43 to 0.80). The ideal item-difficulty levels are ranging between 0.20 and 0.80 (Bühner, 2011). Ideal item-discrimination indices—defined as Pearson product moment correlation between student responses to a particular item and total scores on all other items of the scale—are above 0.30 (see Bühner, 2011). The item-discrimination indices of the German version of the SRQ-A obtained by the pre-tests ranged between 0.35 and 0.74 (intrinsic motivation: 0.52 to 0.74; identified regulation: 0.48 to 0.70; introjected regulation: 0.43 to 0.61; external regulation: 0.35 to 0.57). Sample items of the original version and the presented German translation are presented in **Table 4**.

### Measures

In order to obtain convergent and divergent validity of the German SRQ-A, we chose various questionnaires which are conceptually related to the experience of the basic needs as claimed by SDT. For establishing convergent validity, questionnaires measuring the students' perceived autonomy support, perceived competence, perceived self-efficacy, teacher's reference norms, and joy of learning were used. To evaluate the divergent validity of the scale, questionnaires measuring boredom of learning, anxiety within the school context, and school aversion were implemented.

#### Self-related cognitions

##### School-related perceived self-efficacy questionnaire

The support of students' basic needs by teachers have the strongest impact on their academic motivation (Ricard and Pelletier, 2016). Self-related cognitions such as self-efficacy and student's learning competence play a predictive role for students' competence outcomes (Van Dinther et al., 2011). Therefor self-related cognitions were assessed with the school-related perceived self-efficacy questionnaire (“Schulbezogene Selbstwirksamkeitserwartung”: SWSCH; Jerusalem and Satow, 1999). This questionnaire measures optimistic believes about self-efficacy and self-competencies within a school context; and contains 7 questions, which are rated on a four-point Likert-scale (from *1* = not at all true, to *4* = totally true). Internal consistency was acceptable and ranged between 0.69 and 0.72 for several samples (Jerusalem and Satow, 1999 as cited in Jerusalem et al., 2009, p. 18). Discriminatory power for the items ranged between 0.36 and 0.52 across four different samples (Jerusalem and Satow, 1999 as cited in Jerusalem et al., 2009, p. 18).

##### Perceived competence for learning questionnaire

As mentioned above students performance highly depend on self-related cognitions like students' learning competence and perceived self-efficacy (Van Dinther et al., 2011). Thus, the Perceived Competence for Learning Questionnaire (“Allgemeine Selbstwirksamkeitserwartung”: SWALL; Jerusalem and Schwarzer, 1999, as cited in Jerusalem et al., 2009, p. 14) was used to assess the student's self-perceived level of competence in dealing with difficult academic demands. The questionnaire contains six items that are rated on a four-point Likert-scale (from *1* = not at all true, to *4* = completely true). Internal consistency was acceptable with a Chronbach's α ranging from 0.76 to 0.80 across four different representative samples (Jerusalem and Schwarzer, 1999, as cited in Jerusalem et al., 2009, p. 15). Retest-reliability ranged between rtt = 0.41 for item 1, to and rtt = 0.54 for the items 3 and 7 across four different representative samples.

#### Perceived features of lessons

##### Students' perception of autonomy support questionnaire

Because amotivation arises by controlling teaching behaviors, intrinsic motivation and satisfaction is related to perceived autonomy support (Haerens et al., 2015). Perceived features of the lessons were assessed by the Students' Perception of Autonomy Support Questionnaire (“Selbstbestimmung/Autonomie”: AUTO; Röder and Kleine, 2007, as cited in Jerusalem et al., 2009). This questionnaire evaluates the students' perceived level of autonomy support within a class setting, and contains six items that are rated on a four-point Likert-scale (from *1* = not at all true, to *4* = completely true). Internal consistency was acceptable with a Cronbach's α ranging from 0.71 to 0.76 across four different samples (Röder and Kleine, 2007; as cited in Jerusalem et al., 2009, p.34). Retest-reliability ranged between r_tt_ = 0.41 for the items 2 and 4, and r_tt_ = 0.58 for item 1 across four different representative samples.

##### Perceived teacher reference norm questionnaire

Students' reference norms can impact their performance outcome and reading motivation (Förster and Souvignier, 2014). Thus, the Perceived Teacher Reference Norm Questionnaire (“Schülerperzipierte Lehrerbezugsnormorientierung”: SPLB; Schwarzer et al., 1982, as cited in Jerusalem et al., 2009, p. 26) was administered to assess perceived features of the lessons. The SPLB is a questionnaire that evaluates the students' perception of their teachers' reference norms within a performance context. The questionnaire contains 10 items (see Schwarzer, 1986) that are rated on a four-point Likert-scale (from *1* = not at all true, to *4* = completely true). Schwarzer and Jerusalem (1999, as cited in Jerusalem et al., 2009) reported an acceptable Cronbach's α ranging between 0.74 and 0.78 for a short version, consisting of four items. Retest-reliability ranged between r_tt_ = 0.41 for item 1 and r_tt_ = 0.69 for item 3 across four different representative samples.

#### Students' emotions

##### Joy of learning and boredom questionnaire

Because of teachers supporting autonomy, students experience joy during class and develop a more positive attitude toward learning (Vallerand et al., 1989). In order to assess the students' emotions, two measures were administered: First, the Joy of Learning and Boredom Questionnaire (“Schüleremotionen Lernfreude und Langeweile”: Pekrun, 1993) is a six item questionnaire measuring the students' joy and boredom of learning within an academic context, whereas joy and boredom of learning are evaluated on two different subscales, each containing three items. Each of the items is rated on a four-point Likert-scale (from *1* = not at all true, to *4* = completely true). Internal consistency as measured by Cronbach's α was between 0.65 and 0.72 for the subscale joy of learning, and between 0.77 and 0.78 for the subscale boredom of learning across four different representative samples (Pekrun, 1993 as cited in Jerusalem et al., 2009). Retest-reliability ranged between r_tt_ = 0.40 and r_tt_ = 0.58 for the subscale joy of learning, and between r_tt_ = 0.58 and r_tt_ = 0.64 for the subscale boredom of learning.

##### Anxiety questionnaire for students

Since autonomy supporting teachers enhance students' motivation (Haerens et al., 2015), students experience higher anxiety and therefore less self-efficacy by teachers demonstrating a controlling behavior (Assor et al., 2005). The Anxiety Questionnaire for Students (“Angstfragebogen für Schüler”: AFS; Wieczerkowski et al., 1981) is a multifactorial questionnaire, assessing the students' anxiety provoking and negative experiences within the school context on the basis of four different subscales: Test anxiety, general (manifest) anxiety, school-aversion, and social desirability. The subscale manifest anxiety measures physiological symptoms of anxiety, such as fast heartbeat, nervousness, and difficulties concentrating. The subscale school-aversion evaluates the students' school related defense-mechanisms—such as loss of motivation—caused by aversive experiences within the school context. Internal consistency was reported to be acceptable for subscale school aversion (α = 0.67) and general (manifest) anxiety (α = 0.70) to high for the subscale test anxiety (α = 0.77). For the subscale social desirability no internal consistency was reported by Wieczerkowski et al. (1981). Retest-reliability ranged between r_tt_ = 0.71 (for subscale general (manifest) anxiety) and r_tt_ = 0.76 (for subscales test anxiety and school aversion) after 1 month. There are a total of 50 items subdivided into four subscales. Those items are rated on a six-point Likert-scale (from *1* = not at all true, to *6* = totally true).

### Data analysis

Data analyses was performed using the program RStudio (R version 3.1.0). First, means, standard deviations, skewness, curtosis, and internal consistencies (as reflected by Cronbach's α) of each of the four subscales of the SRQ-A were calculated. Then, correlation analyses were conducted to test the expected simplex structures of the subscales of the SRQ-A (Ryan and Connell, 1989). Moreover, confirmatory factor analyses (CFA) were performed to test the original four-factor model (Ryan and Connell, 1989). Data was separated by gender. Finally, construct validity was evaluated through Pearson correlation coefficients among the four scales of the German version of the 28-item SRQ-A and several indices of self-related cognitions, perceived features of lessons, and students' emotions. In order to avoid an alpha-inflation, results were adjusted for multiple testing according to Bonferroni by dividing 0.05 by four tests, resulting in an α of 0.0125 for significance (0.05/4). In order to determine the relative strength between different correlations coefficients, Steiger's Z test were executed (Meng et al., 1992).

Bartlett's test of sphericity revealed significant results (total sample: *Chi*^2^*(378)* = 9288.47, *p* < 0.001; boys: *Chi*^2^*(378)* = 5118.22, *p* < 0.001; girls: *Chi*^2^*(378)* = 4422.58, *p* < 0.001). Confirmatory factor analyses were performed using maximum likelihood estimation. In the total sample, items were considered to load on the factor if their coefficient was >0.40 (Moilanen, 2007). Due to loadings lower than 0.40, one item (item 6) was dropped from the external subscale and three items (items 4, 29, and 31) were dropped from the introjected subscale. The feasibility of the resulting 28-item model was assessed using several measures—i.e., Chi-square, the Root Mean Square Error of Approximation (*RMSEA*), the Comparative Fit Index (*CFI*), and the Tucker Lewis Index (*TLI*). The Mardia test was significant for all items (either skewness or excess). Therefore, a Bollen-Stine bootstrap correction (1000 samples) of the Chi-square *p*-value was conducted. *RMSEA* values between 0.05 and 0.8 are considered reasonable, and values below 0.05 suggest a good model of fit (Bentler, 1990; Browne and Cudeck, 1993). For both, the *CFI* and the *TLI*, values < 0.90 usually mean that the model can be improved upon substantially (see Bentler and Bonett, 1980).

## Results

### Descriptive statistics, reliabilities, and gender differences

Means, standard deviations, skew, kurtosis, and Cronbach's α of the four subscales of the 28-item SRQ-A are presented according to gender in Table 2. Girls and boys did not differ significantly in external regulation [*t*_(666.72)_ = 1.66, *p* < 0.10] and introjected regulation [*t*_(667.95)_ = 0.02, *p* = 0.98]. Yet, a significant gender difference was found for identified regulation [*t*_(651.47)_ = −3.92, *p* < 0.001] and intrinsic motivation [*t*_(668)_ = −2.81, *p* < 0.01] with girls scoring higher on average than boys. Cronbach's α revealed good levels of internal consistency for all scales of the German version of the 28-item SRQ-A.

**Table 2:** Summary of preliminary analysis by gender.

	**Total Sample (*****n*** = **672)**				**Boys (*****n*** = **344)**				**Girls (*****n*** = **326)**			
	**ER**	**INR**	**IDR**	**IM**	**ER**	**INR**	**IDR**	**IM**	**ER**	**INR**	**IDR**	**IM**
*M*	1.96	1.92	2.33	1.77	1.99	1.92	2.24	1.69	1.92	1.92	2.43	1.85
*SD*	0.59	0.73	0.62	0.76	0.59	0.75	0.67	0.78	0.59	0.70	0.54	0.74
*Min*	0.25	0.00	0.00	0.00	0.25	0.00	0.00	0.00	0.25	0.00	0.43	0.00
*Max*	3.00	3.00	3.00	3.00	3.00	3.00	3.00	3.00	3.00	3.00	3.00	3.00
*Skew*	−0.36	−0.41	−1.16	−0.21	−0.52	−0.50	−1.13	−0.13	−0.20	−0.30	−1.00	−0.27
*Kurtosis*	−0.41	−0.44	1.51	−0.69	−0.19	−0.40	1.29	−0.77	−0.58	−0.52	0.76	−0.60
*Cronbach's* α	0.75	0.82	0.87	0.88	0.75	0.83	0.88	0.88	0.76	0.81	0.85	0.87

*Means (M), standard deviations (SD), minimum (Min), maximum (Max), skewness (SE = standard error), kurtosis (SE = standard error), and Cronbach's α of the four scales external regulation (ER), introjected regulation (INR), identified regulation (IDR), and intrinsic motivation (IM) of the German version of the 28-item SRQ-A. Results are separated according to gender*.

### Simplex structures

Construct validity should be given with all subscales of the SRQ-A being more strongly and positively correlated with those that are theoretically adjacent, than with those that are more distant. As shown by the correlation matrix in Table 3, the simplex structures of the SRQ-A emerged in the present sample.

**Table 3:** Pearson correlation coefficients among the four scales of the German version of the 28-item SRQ-A.

	**External Regulation**	**Introjected Regulation**	**Identified Regulation**	**Intrinsic Motivation**
External regulation	–			
Introjected regulation	0.61	–		
Identified regulation	0.21	0.46	–	
Intrinsic motivation	0.11	0.33	0.74	–

*All correlation coefficients are significantly different from zero at the 0.001 level (two-tailed). Results are adjusted for multiple testing*.

### Confirmatory factor analyses

Table 4 displays the factorial loadings of the individual items of the four scales of the German version of the 28-item SRQ-A. The factorial loadings of the individual items are separated according to gender, testing a four-factor model with three correlations between indicator residual variances. Correlations were specified between items 19 and 22 (total sample: *r* = 0.53, *p* < 0.001; boys: *r* = 0.55, *p* < 0.001; girls: *r* = 0.47, *p* < 0.001), items 3 and 7 (total sample: *r* = 0.34, *p* < 0.001; boys: *r* = 0.36, *p* < 0.001; girls: *r* = 0.29, *p* < 0.001), and items 12 and 18 (total sample: *r* = 0.55, *p* < 0.001; boys: *r* = 0.47, *p* < 0.001; girls: *r* = 0.57, *p* < 0.001).

**Table 4:** Factorial loadings of the SRQ-A by subscale and gender.

	**Total Sample (*n* = 672)**	**Boys (*n* = 344)**	**Girls (*n* = 327)**
**EXTERNAL REGULATION**			
Item 2: Weil ich sonst Ärger bekomme (*Because I'll get in trouble if I don't)*	0.44	0.36	0.52
Item 9: Damit meine Lehrer nicht mit mir schimpfen (*So that the teacher won't yell at me)*	0.64	0.59	0.72
Item 14: Weil es zu den Regeln gehört (*Because that's the rule)*	0.49	0.53	0.43
Item 20: Weil es von mir erwartet wird (*Because that's what I'm supposed to do)*	0.53	0.58	0.46
Item 24: Weil ich möchte, dass meine Lehrer nette Dinge über mich sagen (*Because I want the teacher to say nice things about me)*	0.59	0.58	0.58
Item 25: Weil es von mir erwartet wird (*Because that's what I'm supposed to do)*	0.53	0.63	0.40
Item 28: Weil ich Ärger bekomme, wenn ich nicht gut bin (*Because I will get in trouble if I don't do well)*	0.40	0.37	0.40
Item 32: Weil ich vielleicht eine Belohnung bekomme, wenn ich gut bin (*Because I might get a reward if I do well)*	0.44	0.38	0.49
**INTROJECTED REGULATION**			
Item 1: Weil ich möchte, dass meine Lehrer denken, ich bin ein guter Schüler (*Because I want the teacher to think I'm a good student)*	0.75	0.72	0.78
Item 10: Weil ich möchte, dass meine Lehrer denken, ich bin ein guter Schüler (*Because I want the teacher to think I'm a good student)*	0.72	0.71	0.72
Item 12: Weil ich mich vor mir selber schämen würde, wenn ich sie nicht machen würde (*Because I'll be ashamed of myself if it didn't get done)*	0.48	0.61	0.37
Item 17: Weil ich möchte, dass die anderen Schüler von mir denken, dass ich klug bin (*Because I want the other students to think I'm smart)*	0.69	0.67	0.68
Item 18: Weil ich mich vor mir selber schäme, wenn ich es nicht versuche (*Because I feel ashamed of myself when I don't try)*	0.46	0.56	0.38
Item 26: Damit meine Lehrer denken ich bin ein guter Schüler (*So my teachers will think I'm a good student)*	0.73	0.73	0.72
**IDENTIFIED REGULATION**			
Item 5: Weil ich das Fach verstehen möchte (*Because I want to understand the subject)*	0.52	0.61	0.40
Item 8: Weil ich das Fach verstehen möchte (*Because it's important to me to do my homework)*	0.66	0.64	0.66
Item 11: Weil ich neue Dinge lernen möchte (*Because I want to learn new things)*	0.60	0.66	0.50
Item 16: Weil es mir wichtig ist, im Unterricht mitzuarbeiten (*Because it's important to me to work on my classwork)*	0.62	0.69	0.52
Item 21: Um herauszubekommen, ob ich es weiß oder nicht (*To find out if I'm right or wrong)*	0.40	0.45	0.33
Item 23: Weil es mir wichtig ist, mich anzustrengen (*Because it's important to me to try to answer hard questions in class)*	0.61	0.67	0.54
Item 30: Weil es mir wichtig ist, mich anzustrengen (*Because it's important to me to try to do well in school)*	0.63	0.70	0.54
**INTRINSIC MOTIVATION**			
Item 3: Weil es mir Spaß macht (*Because it's fun*)	0.76	0.74	0.73
Item 7: Weil ich gerne meine Hausaufgaben mache (*Because I enjoy doing my homework)*	0.77	0.72	0.79
Item 13: Weil es mir Spaß macht (*Because it's fun)*	0.80	0.79	0.81
Item 15: Weil ich gerne im Unterricht mitarbeite (*Because I enjoy doing my classwork)*	0.72	0.75	0.68
Item 19: Weil ich gerne schwierige Probleme löse (*Because I enjoy answering hard questions)*	0.49	0.56	0.44
Item 22: Weil es mir Spaß macht, schwierige Probleme zu lösen (*Because it's fun to answer hard questions)*	0.58	0.63	0.55
Item 27: Weil ich gerne meine Aufgaben im Unterricht gut löse (*Because I enjoy doing my school work well)*	0.66	0.69	0.63

*Factor loadings of the items of the four scales of the German version of the 28-item SRQ-A in the confirmatory factor analyses separated according to gender examining the four-factor model proposed by Ryan and Connell (1989). The items refer to different behavioral categories although same reasons are provided (e.g., item 1 and item 10). All regression weights are significantly different from zero at p < 0.001 (two-tailed)*.

In the overall sample, all items were significant (*p* < 0.001) respective their latent factor, and factorial loadings ranged from 0.40 for item 28 (“*Trouble if not doing well in school*”) to 0.80 for item 13 (“*Fun working on classwork”*; cf., Table 4). The pattern of latent correlations between the four SRQ-A factors (Table 5) was consistent with the simplex pattern proposed by the English SRQ-version. However, there was a poor fit between the data and the assumed four-factor structure (*Chi*^2^*(341)* = 1295.30, *p* < 0.001, *TLI* = 0.895, *CFI* = 0.883, *RMSEA* = 0.065).

**Table 5:** Latent correlations between the four scales of the German version of the 28-item SRQ-A.

	**External Regulation (Boys/Girls)**	**Introjected Regulation (Boys/Girls)**	**Identified Regulation (Boys/Girls)**	**Intrinsic Motivation (Boys/Girls)**
External regulation (boys/girls)	–			
Introjected regulation (boys/girls)	0.80 (0.76/0.86)	–		
Identified regulation (boys/girls)	0.30 (0.41/0.16)	0.42 (0.60/0.23)	–	
Intrinsic motivation (boys/girls)	0.19 (0.28/0.09)	0.33 (0.48/0.20)	0.90 (0.90/0.89)	–

*All latent correlations are significantly different from zero at p < 0.001 (two-tailed). Results are Bollen-Stine Bootstrap correct*.

For the boy subgroup, all items showed significant results (*p* < 0.001) on their latent factor. Factor loadings ranged from 0.36 for item 2 (“*No trouble if homework”*) to 0.79 for item 13 (“*Fun working on classwork”*; cf., Table 4). There was a poor fit between the data and the assumed four-factor structure (*Chi*^2^*(341)* = 963.92, *p* < 0.001, *TLI* = 0.873, *CFI* = 0.859, *RMSEA* = 0.073). In the girl subgroup, all items showed significant results (*p* < 0.001) on their latent factor. Factor loadings ranged from.33 for item 21 (“*Answering hard questions to find out if being right or wrong”*) to 0.81 on item 13 (“*Fun working on classwork”*; cf. Table 4). Again, there was a poor fit between the data and the assumed four-factorial structure (*Chi*^2^ *(341)* = 769.86, *p* < 0.001, *TLI* = 0.898, *CFI* = 0.887, *RMSEA* = 0.062). Considering the factor loadings (cf., Table 4), there were differences according to gender, especially with regards to the scale identified regulation, whereas the factor loadings were lower in the girl subgroup. For both boys and girls, the pattern of latent correlations between the four factors (cf., Table 5) was consistent with the expected simplex pattern (Ryan and Connell, 1989).

### Correlations with indices of self-related cognitions, perceived features of lessons, and students' emotions

Convergent and divergent validity of the hereby translated German SRQ-A were assessed by correlations with indices of self-related cognitions, perceived features of lessons and students' emotions. As presented in Table 6, self-related cognitions, perceived features of lessons, and students' positive emotion joy of learning were positively related to the self-determined types of behavioral regulation. On the continuum of self-determination (from intrinsic motivation to external regulation), the correlations decreased continuously—except for four scales. The scales solicitude of the teachers, perceived autonomy, and autonomy support of the teachers were more strongly associated with the scale identified regulation than with the scale intrinsic motivation. The scale comprehensibility was more strongly related to the scale external regulation than to the scale introjected regulation.

**Table 6:** Pearson correlation coefficients among the four scales of the German version of the 28-item SRQ-A and several indices of self-related cognitions, perceived features of lessons, and students' emotions.

	**External Regulation**	**Introjected Regulation**	**Identified Regulation**	**Intrinsic Motivation**
**SELF-RELATED COGNITIONS**				
**School-related Perceived Self-efficacy**	**0.00**	0.21^***^	0.48^***^	0.51^***^
Steiger's Z				
External regulation	−	−6.19, *p* < 0.001	10.3, *p* < 0.001	10.53, *p* < 0.001
Introjected regulation	−6.19, *p* < 0.001	−	7.32, *p* < 0.001.	7.42, *p* < 0.001
Identified regulation	10.3, *p* < 0.001	7.32, *p* < 0.001.	−	−1.27, *p* < 0.21
Intrinsic motivation	10.53, *p* < 0.001	7.42, *p* < 0.001	−1.27, *p* < 0.21	−
**Perceived Competence for Learning**	**0.09**	0.32^***^	0.62^***^	0.59^***^
Steiger's Z				
External regulation	−	−6.91, *p* < 0.001	−12.32, *p* < 0.001	−10.86, *p* < 0.001
Introjected regulation	−6.91, *p* < 0.001	−	−8.91, *p* < 0.001	−7.16, *p* < 0.001
Identified regulation	−12.32, *p* < 0.001	−8.91, *p* < 0.001	−	1.40, *p* = 0.16
Intrinsic motivation	−10.86, *p* < 0.001	−7.16, *p* < 0.001	1.40, *p* = 0.16	−
**PERCEIVED FEATURES OF LESSONS**				
**Perceived Autonomy Support**	**0.02**	**0.08**	0.15^*^	0.23^***^
Steiger's Z				
External Regulation	−	−1.76, *p* = 0.08	2.69, *p* < 0.01	4.12, *p* < 0.001
Introjected Regulation	−1.76, *p* = 0.08	−	1.76, *p* = 0.08	3.41, *p* < 0.001
Identified Regulation	2.69, *p* < 0.01	1.76, *p* = 0.08	−	−2.93, *p* < 0.005
Intrinsic Motivation	4.12, *p* < 0.001	3.41, *p* < 0.001	−2.93, *p* < 0.005	−
**Perceived Teacher Reference Norm**	**0.07**	0.19^***^	0.38^***^	0.34^***^
Steiger's Z				
External Regulation	−	−3.54, *p* < 0.001	6.64, *p* < 0.001	5.41, *p* < 0.001
Introjected Regulation	−3.54, *p* < 0.001	−	5.00, *p* = 6.05	3.52, *p* < 0.001
Identified Regulation	6.64, *p* < 0.001	5.00, *p* = 6.05	−	1.55, *p* < 0.12
Intrinsic Motivation	5.41, *p* < 0.001	3.52, *p* < 0.001	1.55, *p* < 0.12	−
**STUDENTS' EMOTIONS**				
**Joy of Learning Subscale**	**0.13**	0.31^***^	0.60^***^	0.66^***^
Steiger's Z				
External Regulation	−	−5.43, *p* < 0.001	−10.93, *p* < 0.001	12.11, *p* < 0.001
Introjected Regulation	−5.43, *p* < 0.001	−	8.49, *p* < 0.001	9.64, *p* < 0.001
Identified Regulation	−10.93, *p* < 0.001	8.49, *p* < 0.001	−	−2.90, *p* < 0.005
Intrinsic Motivation	12.11, *p* < 0.001	9.64, *p* < 0.001	−2.90, *p* < 0.005	−
**Boredom Subscale**	−**0.01**	−0.22^***^	−0.52^***^	−0.55^***^
Steiger's Z				
External Regulation	−	−6.19, *p* < 0.001	−11.19, *p* < 0.001	−11.32, *p* < 0.001
Introjected Regulation	−6.19, *p* < 0.001	−	−8.26, *p* < 0.001	−8.31, *p* < 0.001
Identified Regulation	−11.19, *p* < 0.001	−8.26, *p* < 0.001	−	−1.31, *p =* 0.19
Intrinsic Motivation	−11.32, *p* < 0.001	−8.31, *p* < 0.001	−1.31, *p =* 0.19	−
**Manifest Anxiety (AFS)**	0.27^***^	0.16^**^	−**0.05**	−**0.10**
Steiger's Z				
External Regulation	−	3.31, *p* < 0.001	6.65, *p* < 0.001	7.23, *p* < 0.001
Introjected Regulation	3.31, *p* < 0.001	−	5.24, *p* < 0.001	5.81, *p* < 0.001
Identified Regulation	6.65, *p* < 0.001	5.24, *p* < 0.001	−	1.80, *p =* 0.07
Intrinsic Motivation	7.23, *p* < 0.001	5.81, *p* < 0.001	1.80, *p =* 0.07	−
**School**−**Aversion (AFS)**	**0.12**	−**0.13**	−0.54^***^	−0.57^***^
Steiger's Z				
External Regulation	−	7.31, *p* < 0.001	−14.24, *p* < 0.001	−14.23, *p* < 0.001
Introjected Regulation	7.31, *p* < 0.001	−	−11.11, *p* < 0.001	−10.90, *p* < 0.001
Identified Regulation	−14.24, *p* < 0.001	−11.11, *p* < 0.001	−	1.33, *p =* 0.18
Intrinsic Motivation	−14.23, *p* < 0.001	−10.90, *p* < 0.001	1.33, *p =* 0.18	−

*Pearson correlations between the four scales of the German version of the 28-item SRQ-A and several indices of self-related cognitions, perceived features of lessons, and students' emotions. Results are adjusted for multiple testing using Bonferroni adjusted alpha levels of 0.0125 per test (0.05/4)*.

*Bold values are the correlations of the overall questionnaire scores with each of the SRQ-A subscales*.

* *The corresponding correlation coefficient is significantly different from zero at p < 0.0125 (two-tailed)*.

** *The corresponding correlation coefficient is significantly different from zero at p < 0.001 (two-tailed)*.

*** *The corresponding correlation coefficient is significantly different from zero at p < 0.0001 (two-tailed)*.

*A test for significance between the four scales of the German version of the 28-item SRQ-A scales was conducted with Steigers's Z Test*.

Regarding the students' emotions (cf., Table 6), boredom was negatively associated with the autonomous types of behavioral regulation. On the continuum of self-determination (from intrinsic motivation to external regulation), the correlation decreased continuously. While the emotion manifest anxiety was negatively related to the controlled types of behavioral regulation, the emotion school reluctance was negatively associated with the self-determined types of behavioral regulation.

Steiger's Z tests were performed to determine the relative strength of the correlations between the four subscales of the German version of the SRQ-A and the indices of self-determined cognitions, perceived features of lessons and students' emotions (see Table 6). As expected, the observed correlations with more autonomous self-regulated motivations styles such as intrinsic motivation and identified regulation were significantly stronger than those depicted for more externally regulated motivation styles such as external motivation and introjected motivation.

## Discussion

The aim of the present study was to evaluate the utility of the German version of the SRQ-A as a self-report measure for self-determination motivation styles, by reporting psychometric properties, examining the factorial structure of the SRQ-A, assessing construct validity in a large sample of primary and junior high school children, as well as gender differences.

The results of the present study indicate good levels of internal consistency for all subscales of the German version of the SRQ-A. Those levels are comparable to the original version of the SRQ-A (Ryan and Connell, 1989). Manifest as well as latent correlations between the four SRQ-A subscales reveal a pattern consistent with the continuum of self-determination, where theoretically adjacent subscales have stronger positive correlations than more distant subscales (Ryan and Connell, 1989). Four items were excluded due to low loadings on their expected latent factor. The final German questionnaire therefore resulted in 28 items, whereas the original English version excluded two more items, because of insufficient variability. The 28 items in the German questionnaire show significant loadings on their expected latent factor.

The assumed four-factor model cannot be replicated in the German sample of primary and junior high school children using confirmatory factor analysis. As previously seen, confirmatory factor analytical approaches were also used by Ryan and Connell (1989), resulting in a two-factorial model with opposing ends defined as internal and external. Even though the authors acknowledged a good discriminant validity, they indicated that if they would accept this two-factorial model, then “meaningful psychological categories would fail to be considered because of the procrustean bed of this factor analytic approach” (Ryan and Connell, 1989, p. 753), therefore assuming a four-factorial model. This assumption could also be applied to the German version of the SRQ-A.

The current study analyzed the presented data including the factor gender, and revealed significant differences between boys and girls for the factors identified regulation and intrinsic motivation, with girls scoring higher on average. Previous studies have evaluated gender differences in the context of basic needs and self-determined motivational styles, revealing rather inconsistent results (e.g., Grolnick and Ryan, 1990; Deci et al., 1992). However, in contrast to the original version of the SRQ-A (Ryan and Connell, 1989), which did not evaluate gender differences, the here presented results are aligning with the Italian validation of the SRQ-A, which also found gender invariances for their translated SRQ-A questionnaire (Alivernini et al., 2011). Additionally, confirmatory factor analysis revealed gender differences between factorial loadings for boys and girls, with lower loadings for girls on the identified factor. Therefore, it might be worthwhile for future studies to consider creating different scoring manuals for boys and girls.

Comparing the results of the current validation of the SRQ-A to the validation presented by Müller et al. (2007) there are important differences. Although, the four factorial as well as simplex structure of the model was also confirmed by Müller et al. (2007); and the measurement demonstrated good internal consistencies, the questionnaire itself is vastly different than the here translated version of the original SRQ-A (Ryan and Connell, 1989). After running CFA in the present study, four items were excluded from the original SRQ-A items, due to low loadings on the respective factor. However, the study by Müller et al. (2007) implemented only nine out of 26 items from the original SRQ-A in their German version, while also adding several items from other scales such as the Academic Motivation Scale (Vallerand et al., 1992), resulting in a 16-item questionnaire that is not an equivalent translation of its original English counterpart. Secondly, the present study validates the German SRQ-A for primary and secondary school children, starting at the ages 8 to 14, whereas the study by Müller et al. (2007) only takes adolescents at the ages 10 and up into account. Therefore, comparisons between the two studies should be made with caution.

Ryan and Connell (1989) determined external validity by using three questionnaires, assessing intrinsic and extrinsic motivation in classroom (Harter, 1981), perceived classroom contexts (autonomy vs. control; DeCharms and Shea, 1976) and perceived internal/external control over outcomes (Ryan et al., 1985). According to the present study, they also reported graded series of correlations on the continuum of self-determination (from intrinsic motivation to external regulation) and suggested, the similarity with external criteria increase by the proximity of the subscales. The present study established the validity of the SRQ-A by correlating its scores to substantiated measures evaluating self-related cognitions, perceived features of lessons, and students' emotions. As expected, the questionnaires for self-related cognitions and perceived features of lessons correlated the most with the subscales intrinsic and identified motivation, indicating good convergent validity and accord with Ryan and Connell (1989) results that mastery motivation as a broader concept of intrinsic motivation (Harter, 1981) correlated the most with the subscales identified and intrinsic motivation. However, questionnaire items that were associated with autonomy and teacher based evaluations, displayed higher associations with the subscale identified regulation. This is not surprising, considering that all of those factors include some sort of external component, and are not solely internally controllable and are also reported by the origin study (Ryan and Connell, 1989), whereas the questionnaire for perceived control over outcomes also indicated high convergent validity with identified regulation. Looking at the students' emotions, the correlational results were aligning with the proposed theory and previous findings (Ryan and Connell, 1989). Whereas in the present study positive aspects of learning, such as joy, were strongly positively related to intrinsic motivation, and negative aspects, such as boredom, anxiety, or school reluctance, were negatively associated with intrinsic motivation, indicating good discriminant as well as convergent validity of the SRQ-A. Ryan and Connell (1989) assessed students' self-reported coping strategies and correspond to the pattern as positive coping strategies proposed convergent validity with the non-external motivational category.

Previously, the SRQ-A has most widely been used within a scholastic environment, in order to evaluate factors that might correlate with the students' motivation. These factors include, but are not limited to, parenting styles (e.g., Grolnick and Ryan, 1989), parental support and academic performance (Grolnick et al., 1991), engagement and performance (Miserandino, 1996), and perceived control and emotion (Patrick et al., 1993). The German version of the SRQ-A could therefore be similarly implemented within the academic context, specifically within primary and secondary schools, in order to measure factors that might influence the students' motivation in a scholastic environment, such as parental or teacher influences, or specific traits and states within the student that might correlate with their motivational style.

## Limitations

The present version of the German SRQ-A has been validated in an academic context with students attending the grades three to six. Thus, its application to school starters is restricted as a third grade reading level is mandatory. Also, the German questionnaire needs further evaluation with students attending higher secondary grades or even college or university level. In addition, the German SRQ-A is a self-report questionnaire and hence, is suspect to various self-distortions in perception, specifically when it comes to vulnerable emotions. It is indicated for future studies to investigate teacher and parental reports, which might be useful to gain a more holistic insight into the students' motivational mechanisms.

## Conclusion

The present study conducted with a large representative school children sample demonstrated that the German version of the SRQ-A is a reliable and valid self-report instrument for the assessment of self-regulated motivations styles within the scholastic environment even at a primary school level. Furthermore, its psychometric properties are comparable to the original scale. In summary, our findings suggest that the German SRQ-A will be a useful tool within the educational context, as well as for research evaluating self-determined behavioral regulation in educational settings.

## Ethics statement

This study was carried out in accordance with the recommendations of the Internal Review Board of the Medical Faculty of the University of Ulm with written informed consent from all subjects and their respective parents. All subjects gave written informed consent in accordance with the Declaration of Helsinki. The protocol was approved by the Local Internal Review Board of the Medical Faculty of the University of Ulm.

## Author contributions

JK: Study conception and design, analysis and interpretation of data, manuscript writing and editing. CG: Study design, acquisition of data, manuscript editing. CS: Analysis of data, manuscript writing and editing. JS: Analysis of data, manuscript editing. ZS: Study conception and design, interpretation of data, manuscript editing, critical revision.

### Conflict of interest statement

The authors declare that the research was conducted in the absence of any commercial or financial relationships that could be construed as a potential conflict of interest.
